# Epidemiological analysis of asymptomatic SARS-CoV-2 transmission in the community: an individual-based model

**DOI:** 10.1038/s41598-021-84893-4

**Published:** 2021-03-18

**Authors:** Zuiyuan Guo, Dan Xiao

**Affiliations:** 1 Department of Disease Control, Center for Disease Control and Prevention in Northern Theater Command, Shenyang, China; 2 China National Clinical Research Center for Neurological Diseases, Beijing Tiantan Hospital, No. 119, South 4th Ring Road West, Fengtai District, Beijing, China

**Keywords:** Computational biology and bioinformatics, Mathematics and computing

## Abstract

We established an individual-based computer model to simulate the occurrence, infection, discovery, quarantine, and quarantine release (recovery) of asymptomatic SARS-CoV-2 infected individuals or patients within the community. The model was used to explore the effects of control measures, such as active tracing, laboratory testing, active treatment, and home quarantine on the epidemic. Considering the condition that *R*_0_ = 1.2, when a case of an imported asymptomatic infected individual (AII) was reported in the community, the implementation of control measures reduced the number of AIIs and patients by 62.2% and 62.4%, respectively. The number of undetected AIIs and patients peaked at 302 days of the epidemic, reaching 53 and 20 individuals, respectively. The implementation of sustained active tracing, laboratory testing, active treatment, and home quarantine can significantly reduce the probability of disease outbreaks and block the spread of the COVID-19 epidemic caused by AIIs in the community.

## Introduction

In 2020, the coronavirus disease 2019 (COVID-19) pandemic has continued to spread throughout the world^1,2^. According to the World Health Organization statistics, as of April 22, 2020, there have been 2.48 million confirmed cases of COVID-19, including almost 170,000 deaths^2^. In April 2020, China was able to successfully control the spread of the pandemic, and the number of new daily cases has essentially dropped to single digits^3^. Nevertheless, there still are asymptomatic severe acute respiratory syndrome coronavirus 2 (SARS-CoV-2)-infected individuals (AIIs) in the population; as of April 22, 984 AIIs remain under medical observation in China^4^.

In this study, AIIs refer to those who often experience mild, limited, or no pneumonia and hence go unrecognized, but are infected with SARS-CoV-2^5^. Surveillance data from China showed that there is a sustained occurrence of second-generation cases among the close contacts of AIIs, and that AIIs can cause clustered outbreaks^6–12^. Studies with small sample sizes have shown that the viral load in the respiratory tract specimens of AIIs did not differ significantly from those of confirmed cases^6^. Some experts believe that even though pathogenic nucleic acids can be detected in the respiratory tract specimens of AIIs owing to the absence of clinical symptoms, such as coughing and sneezing, they have a lower chance of viral shedding compared with patients who have confirmed cases^6^.

The findings of the above-mentioned studies suggest that AIIs carry transmission risks. The first factor is the covertness of transmission. As AIIs do not exhibit clear pneumonia or signs, it is difficult to detect these individuals in the population, which can lead to problems in preventing transmission. The second factor is the subjectivity of the symptoms. Individuals with mild or atypical pneumonia may think that they have not been infected with SARS-CoV-2. Hence, they will not take the initiative to seek medical help and are difficult to detect in routine clinical work. The third factor is the limitations of detection. Because of the presence of a testing window period, it is difficult to identify all AIIs using nucleic acid and serological tests. The existing AIIs in China have been primarily identified through active screening of individuals who had close contact with confirmed cases, retrospective investigation of infection causes among patients, screening of individuals exposed to clustered outbreaks, and active testing of personnel in high-risk areas. However, identifying AIIs remains a challenge^13^.

A number of epidemiological studies evaluating patients with COVID-19 have been published^14–17^, while only a few studies have been conducted on AIIs^18^. As AIIs experience mild or no pneumonia, it is impossible to include the discovery and isolation of AIIs as one of the leading measures for pandemic prevention and control in clinical practice^13^. Therefore, some AIIs are not usually included in the prevention and control of the pandemic, thus, increasing the difficulty and uncertainty in identifying and managing this group. Questions, such as the role played by AIIs in pandemic transmission, whether AIIs can cause the sustained presence of COVID-19 in the population, and whether the management measures for AIIs adopted by China can effectively curb the spread of the pandemic are all of great significance for a more profound understanding of the transmission mechanisms underlying the COVID-19 pandemic and for guiding the government in taking more precise measures to prevent and control the pandemic.

Based on the epidemiological characteristics of AIIs, an individual-based stochastic computer model was established in this study to simulate the spread of the epidemic within the community, which incorporated some of the prevention and control measures adopted by China in response to AIIs, to quantitatively analyze the impact of AIIs on the development trends of the pandemic and to theoretically evaluate the effectiveness of the interventional measures. In addition, we would like to explore consistent solution programs during a regular pandemic as well as emergency prevention and control measures in case of an outbreak because in the real world, non-special and urgent strict control measures may be the normal state of society.

## Methods

### Preconditions

The following preconditions were specified for model establishment: 1. The outbreak occurred within a closed community of 3300 households and approximately 10,300 residents. The number of family members varied from one to seven, and followed a Poisson distribution with a mean of 3. Within the study period, no births (immigration) and deaths (emigration) were reported in the community, and the residents were generally susceptible to SARS-CoV-2. 2. All AIIs and patients underwent the incubation period, infectious period, and recovery period. No group was infectious during the incubation and recovery periods, and viral nucleic acid could not be detected in the respiratory tract specimens. Both groups were infectious during the infectious period, and viral nucleic acids could be detected in respiratory tract specimens. 3. Patients who were diagnosed were quarantined in the hospital to receive appropriate treatment. AIIs who were discovered were quarantined at home for 14 days. None of the current studies have provided a definite period in which the AIIs become infectious; hence, the infectious period of AIIs was determined based on the symptomatic period of COVID-19 patients. Further, once patients and AIIs were confirmed, all their family members were regarded as close contacts and were quarantined for a maximum of 14 days, after which they were allowed to move freely. 4. AIIs and patients developed immunity after prolonged exposure to the virus and could not be re-infected with SARS-CoV-2. 5. The event involving the transmission from AIIs or patients to susceptible persons during the infectious period is a Poisson process with a rate of $\lambda$ (where $\lambda$ is the basic reproduction number divided by the infectious period). The above parameter values and their statistical distributions are shown in Table 1.

**Table 1:** Model parameters.

Description	Distribution characteristics	Numerical values	Sources
Basic reproduction number of AIIs (*R*_0_)	Constant	0.8, 1.2, 1.6	Assumed
Basic reproduction number of patients (*R*_1_)	Uniform distribution	4–5	^21^
Probability of newly infected persons developing into patients (*p*)	Bernoulli distribution	0.2	Inferred according to^22^
Probability of tracing the infection sources of individuals who were in close contacts with the patients (*q*)	Bernoulli distribution	0.5	Assumed
The duration from the time the patient first sought medical attention until the time their infection source or close contacts have been traced ($\tau$)	Uniform distribution	1–2	Assumed
Infectious period of AIIs	Uniform distribution	15–20	Inferred according to^23^
Incubation period of AIIs and patients	Lognormal distribution	$\mu = 5.2$ $\sigma = 0.87$	^24^
Time from disease onset to seeking medical attention	Weibull distribution	$\mu = 4.6$ $\sigma = 0.26$	^24^
Number of family members	Poisson distribution	$\mu = 3$	Assumed

### Simulation

#### Infection

AIIs and patients became sources of infection after an incubation period. They transmitted the virus to susceptible persons through daily contact (referred to as contact that can lead to infection) with unquarantined residents in the community. We specified 6:00–18:00 each day as the time frame during which infection sources could randomly come into contact with unquarantined residents (including members of their own families), whereas between 18:00 and 6:00 the next day, infection sources could only randomly come into contact with their own family members. Once a susceptible person has been infected, they have a probability *p* of developing into a patient, with a basic reproduction number *R*_1_, and a probability 1-*p* of developing into an AII, with a basic reproduction number of *R*_0_.

#### Discovery

Patients were admitted to the hospital to receive treatment after the onset of an illness, and their respiratory tract specimens were collected by physicians to test for SARS-CoV-2. Once the patients were diagnosed, the health department traced their infection sources and close contacts through epidemiological surveys. The infection sources and close contacts had a probability *q* of being traced; after that, they underwent laboratory testing for SARS-CoV-2. If the test results were negative, they were excluded. As viral nucleic acids cannot be detected in patients and AIIs during the incubation and recovery periods, a proportion of people will remain undiagnosed.

#### Isolation

For cases where the infection sources or close contacts tested positive, those who showed pneumonia but had not received treatment were considered confirmed cases and were immediately sent to the hospital for quarantine and treatment. Those who were yet to show pneumonia were regarded as AIIs and quarantined at home and put under observation. In addition, all family members were also quarantined at home for 14 days from the time the confirmed cases and AIIs were identified. During home quarantine, if another COVID-19 patient or AII was discovered among the family members, those family members will be quarantined for another 14 days on the day of discovery. During home quarantine, none of the family members were allowed to come in contact with other residents, and only one of them was allowed to purchase their daily necessities while wearing a mask. If someone in the household was confirmed after the quarantine was lifted, then home quarantine was once again imposed based on the strategy described above. For the computer program used in the “Methods” section, please refer to the supplemental material.

### Sensitivity analyses

We performed sensitivity analyses of five significant parameters to assess the impact on the attack rate. Partial rank correlation coefficients (PRCCs) and Latin hypercube sampling (LHS) were used when performing sensitivity analyses. PRCC-LHS is an efficient and reliable sampling-based sensitivity analysis method that measures the monotonicity between a set of parameters and the model output after the removal of the linear effects of all parameters except the parameter of interest^19,20^. Each parameter interval was divided into *N* smaller and equal intervals, and one sample was selected randomly from each interval^19,20^. A standard coefficient denoting the correlation between the parameter and the model output was calculated. All analyses were conducted using MATLAB R2019a (MathWorks, USA, 2019).

We studied the characteristics of the COVID-19 epidemic spread in a virtual environment, which is a theoretical simulated epidemiological model study. The research data are simulated by the computer, without using the real-world population survey database. The parameter values required to establish the model are partly derived from the author's inference and partly from the literature, which are open-source data (See Table 1 for details).

## Results

### Epidemic caused by one case of imported AII

#### Probability of community outbreak and incidence among residents

**Figure 1:**
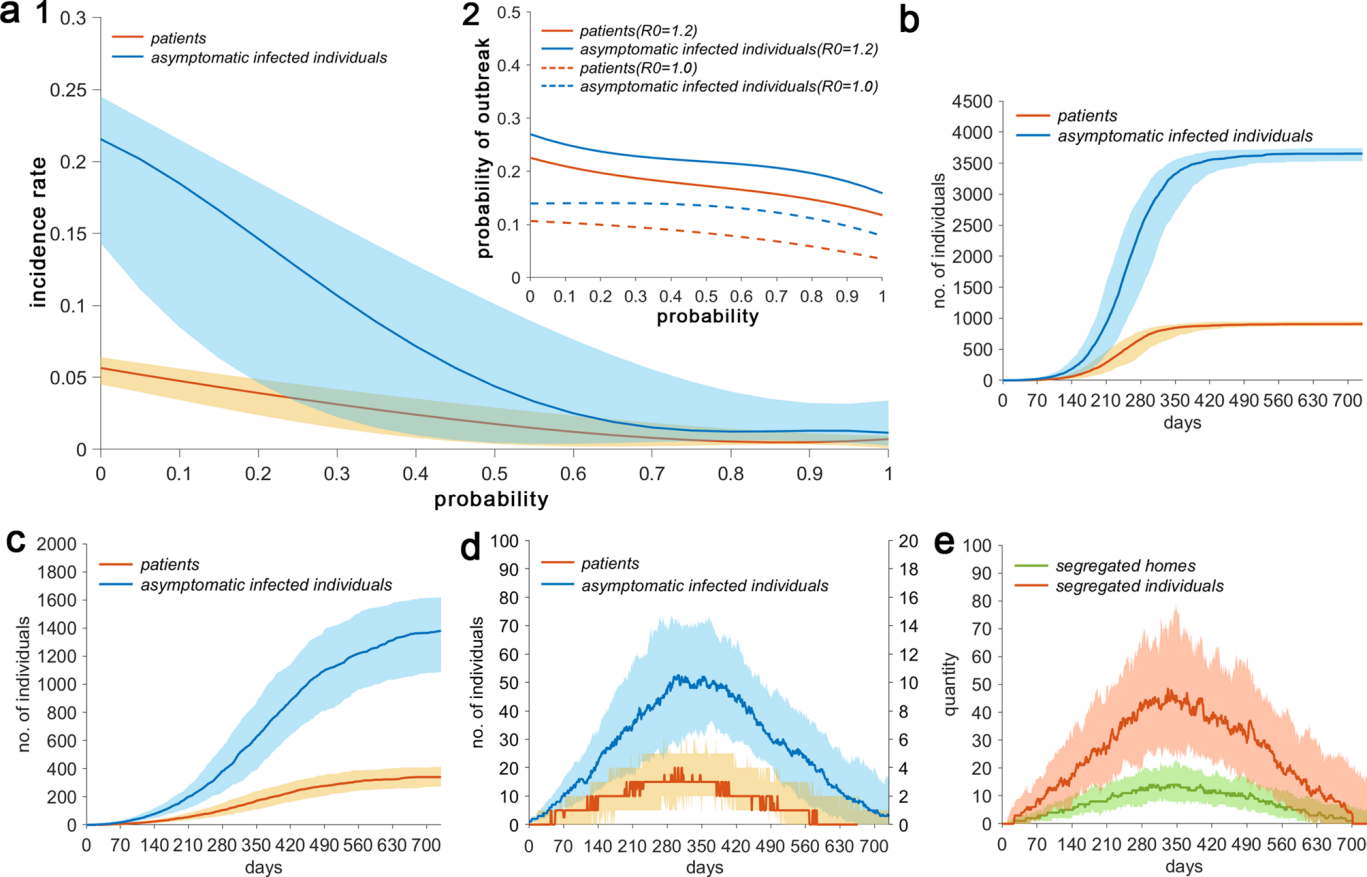
Transmission of an epidemic caused by one case of imported AII. (**a1**) Considering *R*_0_ = 1.2, *p* = 0.2, *t* = 730 days, and in the presence of prevention and control measures, the effect of parameter *q* on the infection rate of AIIs, and incidence rate of patients during an epidemic outbreak (cumulative number of new AIIs more than 20 or cumulative number of new patients more than 10). (**a2**) Considering *R*_0_ = 1.2, 1.0, and in the presence of measures, the effect of parameter *q* on the probabilities of AIIs and patients causing an epidemic outbreak. (**b**) Considering *R*_0_ = 1.2, and in the absence of any measures, the temporal distribution of the cumulative number of new AIIs and patients during an epidemic outbreak. (**c**) Considering *R*_0_ = 1.2, and in the presence of measures, the temporal distribution of the cumulative number of new AIIs and patients during an epidemic outbreak. (**d**) Considering *R*_0_ = 1.2, and in the presence of measures, the temporal distribution of the number of undetected AIIs and patients. (**e**) Considering *R*_0_ = 1.2, and in the presence of measures, the temporal distribution of the number of quarantined households and residents during an epidemic outbreak.

Outbreak was defined as the occurrence of more than ten patients or more than 20 AIIs within the community. There were 3300 households within the community and approximately 10,300 residents. Figure 1a1 shows that when *R*_0_ = 1.2, *p* = 0.2, and *q* = 0, and under the conditions of an outbreak, the median infection rate of AIIs and median incidence rate of patients at 730 days of the epidemic were 0.22 and 0.06, respectively. As *q* increased, the infection rate and incidence rate decreased. When *q* = 1, the medians decreased to 0.01 and 0.005, respectively. Figure 1a2 shows that compared with *R*_0_ = 1, when *R*_0_ = 1.2, the probability of patients and AIIs to cause an outbreak increases.

#### Temporal distribution of the cumulative number of new AIIs and patients in the absence of interventions

Figure 1b shows that without the tracing of infection sources and close contacts and without the home quarantine of AIIs, considering the premise of a community outbreak, the cumulative number of AIIs and patients during a community outbreak increased from almost 0 to 3653 (25–75% percentile [50% P]: 3529–3741) and 907 (50% P: 875–949), respectively.

#### Epidemic trends in the presence of interventions

Figure 1c shows that if the tracing of infection sources and close contacts and the home quarantine of AIIs after detection were implemented, considering the premise of a community outbreak, the number of AIIs and patients increased to 1381 (50% P: 1092–1620) and 340 (50% P: 272–414), respectively, when *t* = 730. In the absence of interventions, the medians of the two groups decreased by 62.2% and 62.5%, respectively. Figure 1d shows that the number of undetected AIIs and patients initially increased, but eventually decreased to 0. The proportion of AIIs and COVID-19 patients peaked at 302 days, reaching 53 (50% P: 27–71) and 20 individuals (50% P: 10–25), respectively. Moreover, Fig. 1e shows that the number of quarantined individuals and households initially increased, but eventually decreased to 0. The peaks of the two groups appeared at 333 days, reaching 49 individuals (50% P: 26–65) and 14 households (50% P: 6–21), respectively.

### Epidemic caused by 1000 AIIs in the presence of interventions

#### Temporal distribution of the number of new AIIs and patients

**Figure 2:**
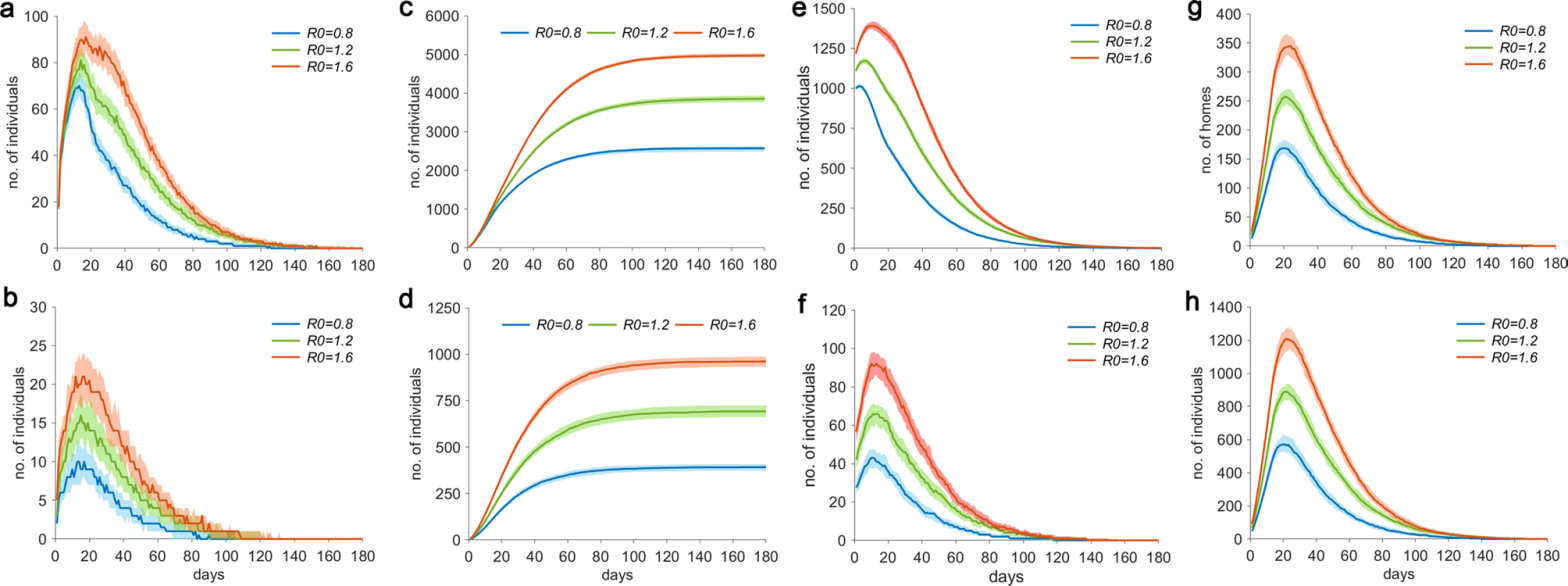
Considering *p* = 0.2, *q* = 0.5, an initial number of 1,000 AIIs, and in the presence of prevention and control measures, the transmission characteristics of the epidemic for different values of *R*_0_. (**a**) Temporal distribution of the number of new AIIs. (**b**) Temporal distribution of the number of new patients, (**c**) Temporal distribution of the cumulative number of new AIIs, (**d**) Temporal distribution of the cumulative number of new patients, and (**e**) Temporal distribution of the number of undetected AIIs. (**f**) Temporal distribution of the number of undetected patients. (**g**) Temporal distribution of the number of quarantined households. (**h**) Temporal distribution of the number of quarantined residents.

Figure 2a shows that considering the conditions of *p* = 0.2 and *q* = 0.5, when *R*_0_ was 0.8, 1.2, and 1.6, the number of newly infected persons peaked at 13, 14, and 17 days, respectively, reaching 70 (50% P: 66–76), 81 (50% P: 74–87), and 91 individuals (50% P: 86–97). Figure 2b shows that the number of patients peaked at 13, 15, and 16 days, reaching 10 (50% P: 7–13), 16 (50% P: 13–19), and 21 individuals (50% P: 17–24), respectively.

#### Temporal distribution of the cumulative number of new AIIs and patients

Figure 2c shows that when *R*_0_ values were 0.8, 1.2, and 1.6, the cumulative number of newly infected persons at 180 days of the epidemic reached 2573 (50% P: 2491–2624), 3854 (50% P: 3766–3933), and 4971 individuals (50% P: 4918–5027), respectively. Figure 2d shows that the cumulative number of new patients at 180 days of the epidemic reached 392 (50% P: 373–408), 692 (50% P: 661–724), and 962 individuals (50% P: 933–986), respectively.

#### Temporal distribution of the number of undetected AIIs and patients

Figure 2e shows that when *R*_0_ was 0.8, 1.2, and 1.6, the number of undetected AIIs in the infectious period peaked within 3, 7, and 10 days of the epidemic, reaching 1013 (50% P: 1001–1029), 1173 (50% P: 1150–1188), and 1391 individuals (50% P: 1370–1416), respectively. Figure 2f shows that the number of undetected patients in the infectious period peaked at 10, 11, and 13 days of the epidemic, reaching 43 (50% P: 38–47), 66 (50% P: 60–71), and 92 individuals (50% P: 86–97), respectively.

#### Temporal distribution of the number of quarantined households and residents

Figure 2g shows that when *R*_0_ values were 0.8, 1.2, and 1.6, the number of quarantined households peaked at 20, 21, and 24 days of the epidemic, reaching 169 (50% P: 160–184), 258 (50% P: 244–269), and 345 households (50% P: 325–362), respectively. Figure 2h shows that the number of quarantined residents peaked at 20, 21, and 22 days of the epidemic, reaching 571 (50% P: 525–628), 888 (50% P: 838–924), and 1,208 individuals (50% P: 1130–1272), respectively.

### Sensitivity analyses

**Figure 3:**
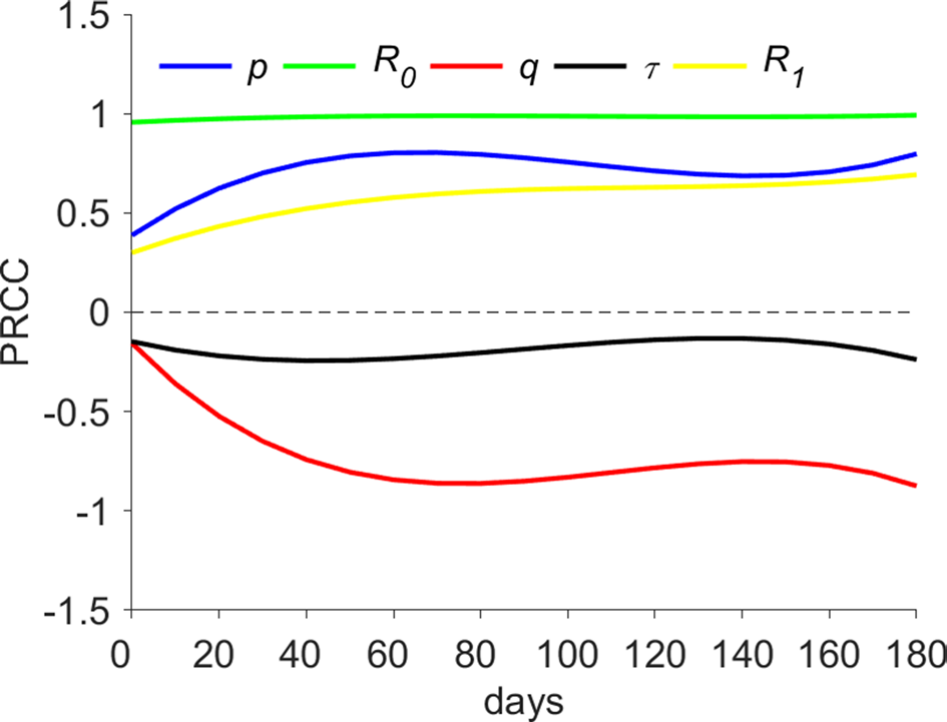
Results of continuous-time sensitivity analyses.

In this study, sensitivity analyses of the five parameters and a continuous time series of the sum of asymptomatic infected individuals and patients each day were conducted. We obtained 500 samples from a uniform distribution for each parameter range. PRCCs near –1 or + 1 indicate that the parameter has a strong negative or positive impact on the output, respectively, whereas those closer to 0 indicate less effect on the output result for that parameter (Fig. 3). The results indicated that *R*_0,_ *R*_1,_ and *p* had a positive effect on the model outputs; meanwhile, $\tau$ and *q* had a negative effect on the model outputs.

## Discussion

The advantages of this study are mainly manifested in the following aspects: First, the individual-based computer model combines the covertness and infectivity of AIIs who underwent preventive and control measures, such as tracing, treatment, and quarantine, which made the model more compatible with the actual conditions of the epidemic prevention and control. Second, the model randomly assigns the parameters for the daily activities and disease characteristics of residents as well as medical treatment according to their respective statistical distributions, which accords the model richer and more powerful analytical capabilities compared with the traditional dynamic models. Thus, the results of the model will provide more precise guidance for the government’s undertakings in preventing and controlling the disease. Third, there is a current absence of epidemiological studies on AIIs; therefore, using computer simulations to quantitatively analyze the epidemiological distribution of AIIs and patients in the population, we have provided a preliminary solution to address the international concerns of coping with AIIs.

The results in Fig. 1a show that when one case of imported AII is reported in a community, the probability of an epidemic outbreak decreases with the increase in parameter *q*. This finding reflects the fact that tracing the infection sources and close contacts of patients not only enables the early discovery of AIIs and patients in the community, but also lays the foundation for the timely isolation of AIIs at home and the isolation of patients for treatment. This, in turn, will reduce the probability of epidemic outbreaks and the incidence rates among residents. In addition, the probability of outbreak is related to the infection time of the imported AII; that is, the earlier the infection time, the shorter the infectious period in the community, and the lower the probability of an epidemic outbreak. The model randomly assigns the infection time, which ensures the objectivity of the results to a certain extent.

The results in Fig. 1b,c show that in the absence of any intervention measures, where AIIs and patients are allowed to transmit the disease in the community, approximately 44% of residents will be infected with SARS-CoV-2 in the event of an outbreak. In the presence of intervention measures, that is, though the infection sources and close contacts of patients have a 50% chance of being discovered, the proportion of infected residents decreased by 62% in the event of an outbreak compared with the absence of such control measures. This finding once again confirms that prevention and control measures can significantly reduce the risk of infection among residents.

Undetected AIIs and patients are the primary sources of infection during an epidemic, and understanding the temporal distribution characteristics of their numbers is of significant value for predicting epidemic trends. Furthermore, determining the number of quarantined individuals and households will facilitate the government’s rational allocation of livelihood materials and safeguard the normal daily routine of quarantined individuals. The results in Fig. 1d,e show that the number of undetected AIIs and patients as well as the number of quarantined residents and households initially increased, but eventually decreased to 0. This finding indicates that as long as the normalization of such control measures is maintained, the epidemic will eventually be controlled.

The results in Fig. 2 show that the number of new and undetected AIIs and patients as well as the number of quarantined households and individuals showed a rapid initial growth, followed by a slow decline to 0. The primary cause for this trend is that the early stage of the outbreak is dominated by the community transmission of SARS-CoV-2 by patients and AIIs. With the continuous tracing of patients and AIIs for quarantine, the number of freely moving infection sources will not only reduce, but the transmission routes of the virus will also be eliminated, thereby gradually controlling the epidemic.

The results of the sensitivity analyses showed that the PRCCs of *R*_0_ remained close to 1, indicating that the basic reproduction number of AIIs has a substantial impact on the total number of infected persons. The PRCCs of *R*_1_ were slightly lower than those of *R*_0_. This may be because although *R*_1_ was greater than *R*_0_, patients accounted for a smaller proportion of infected persons compared with AIIs, which restricted the contribution of the number of patients to the growth in the number of infected persons. In addition, a larger *p* value resulted in a greater number of patients; hence, patients with greater infectivity had an increased number of infected persons. As *q* increased, the number of AIIs and patients detected increased, which reduced the number of newly infected persons. The length of tracing time $\tau$ had a relatively small effect on the total number of infected persons.

Study limitations are mainly manifested in the following aspects: First, the model does not consider the investigation of individuals exposed to clustered outbreaks. This is because the design of this model is not suitable for the simulation of clustered outbreaks, and a new model must be established for such analyses. Second, this model does not consider the active screening of key populations. This is because most countries do not conduct mandatory pathogenic testing of their populations; hence, this program is not universally implemented. Third, AIIs are still infectious during home quarantine and will infect their family members. Therefore, appropriate control measures must be adopted in accordance with the national conditions of each country. Lastly, owing to constraints on computing power, this model is limited to a closed community. However, during the pandemic, most countries and regions have adopted policies, such as restricting movement across borders, and strict home quarantine. Therefore, most regions can be considered to be relatively closed, and the results of this study are applicable to areas with pandemic outbreaks where restrictions on population movement and personnel activities have been imposed.

## Supplementary Information

Supplementary Information
