# Supplementary material for: Epidemiological analysis of asymptomatic SARS-CoV-2 transmission in the community: an individual-based model
Source: Sci Rep. 2021 Mar 18;11:6251. doi: 10.1038/s41598-021-84893-4 (PMC7973571; doi:10.1038/s41598-021-84893-4)
Supplement: Supplementary file 1 — Supplementary Information [file 41598_2021_84893_MOESM1_ESM.pdf]

Supplemental material for epidemiological analysis of asymptomatic SARS-CoV-2 transmission in the community: an individual-based model

Zuiyuan Guo[1], Dan Xiao[2,*]

When the initial value is 1,000 asymptomatic coronavirus disease 2019-infected individuals (AIIs), we used MATLAB R2019a to calculate the time distribution of undetected AIIs and patients in the community.

```
clear all
%%%%%%%%Setting initial values
nhome=3300; nsick=1000; times=500; day=200;     % "Times" represent the number of simulations.
p=0.2;                                          % Probability of onset
CR0=[0.8,1.2,1.6];                              % Three different values of R0
q=0.5;                                          % Probability of detection of close contacts or infectious sources
paicha=1+rand(1,nhome);                         % Duration of the investigation
R1=rand(1,nhome)+4;                             % R1 confirms the uniform distribution from 4 to 5.
infect0=rand(1,nhome)*5+15;                     % The infection period of AIIs
%%%%%%%Assigning the number of individuals in every family
qfq=random('Poisson',3,1,nhome);                % The vector "qfq" is used to record the number of individuals in every family.
f=find(qfq>7 | qfq==0);
while length(f)~=0
        qf=random('Poisson',4,1,length(f));
        qfq(f)=qf;
        f=find(qfq>7 | qfq==0);
end
%%%%%%% Incubation period
```

```
OK=makedist('Loglogistic','mu',1.357,'sigma',0.764);        % Establishing the probability density
                                                            function of Log-logistic
incubation=random(OK,1,nhome);                              % The vector “incubation” was used to
                                                            record the incubation period.
f=find(incubation>14 | incubation<1);
while length(f)~=0
        qf=random(OK,1,length(f));
        incubation(f)=qf;
        f=find(incubation>14 | incubation<1);
end
med=4.1+(5.1-4.1)*rand(1,nhome);          % Vector “med ”is used to store the time from disease onset
                                           to the time that the individual sought medical attention.
Storei=[]; Storep=[];                      % “Storei” and “Storep” are used to store the number of
                                              AIIs and patients, respectively.
%%%%%%%%%%%%%%%%%%%%%%%%%%%%%%%%%%%%%%%%%%%%
for ri=1:length(CR0)
    R0=CR0(ri);
    Infections=[]; Patients=[];      % “Infections” and “Patients” are used to record the number of AIIs and
                                       patients identified from every circulation
    for xh=1:times
      [ri, xh]
      Asy=[]; Pat=[];
      %%%%%%%%%% Establishing the matrix “Home”
      %The meaning of every column of the matrix “Home”: 1. ID, 2. the number of individuals in every
        family, 3. the number of patients, 4. the number of AIIs, 5. the number of current infected individuals,
        and 6. every family member (AIIs, “10”; patients, “20”).
      Home=[];
      Home(:,1)=1:nhome;
      Home(:,2)=qfq;
      Ra=randperm(nhome);
```

```
Ra1=Ra(1:nsick);
Home(Ra1,4)=1;
for i=1:nhome
    Home(i,(6:(Home(i,2)+5)))=[1:Home(i,2)];
end
randsel=round(rand(nsick,1).*Home(Ra1,2)+0.5);    % Randomly selecting infected family members
for i=1:nsick
    Home(Ra1(i),(randsel(i)+5))=10;                % Locations of the AIIs in the family
    Home(Ra1(i),5)=randsel(i);            % The current infected individuals were located in column 5
end
HB=zeros(nhome,21);
HB(:,1)=1;
HA=zeros(1,sum(qfq));                                  % Record the times of isolation
%%% Initializing the matrix "A" that records the spread of the epidemic. The meaning of every
  column in A is as follows: 1. ID, 2. source of infection, 3. the ID of family, 4. "0" represents an AII
  and "1" represents a patient, 5. the basic reproductive number, 6. the time of infection, 7. incubation
  period, 8. time of starting infection period, 9. infection period, 10. the time in which all AIIs and
  patients were detected, 11. storing the time of the next infection, 12. storing the number of infected
  individuals, and 13. the time when susceptible persons were infected
A=[];
A(:,1)=1:nsick;
A(:,2)=0;
A(:,3)=Ra1;
A(:,4)=0;
A(:,5)=R0;
A(:,7)=incubation(unidrnd(nhome,1,nsick));
A(:,6)=-rand(nsick,1).*infect0(unidrnd(nhome,nsick,1))'-incubation(unidrnd(nhome,nsick,1))';
A(:,8)=A(:,6)+A(:,7);
A(:,9)=infect0(unidrnd(nhome,nsick,1));
A(:,10)=A(:,8)+A(:,9);
```

```
Exp=[];
for ei=1:nsick
        ex=exprnd(infect0(unidrnd(nhome))/R0);
        Exp(ei)=ex;         % When the period of initial infection is longer than the period of treatment
end
AE=A(:,8)+Exp';
find1=find(AE>A(:,10));
A(find1,11)=day+1;
A(find1,12)=0;
find2=find(AE<=A(:,10));% When the period of initial infection is shorter than the period of treatment
A(find2,13)=AE(find2);
A(find2,11)=A(find2,13);
A(find2,12)=ones(length(find2),1);
suma=sum(A(:,11)<day);
lengthA=size(A,1);
while suma>0                                % As long as the infection exists before the "day"
                                              indicated in matrix "A," the circulation will continue.
     F=find(A(:,11)==min(A(:,11)));          % Detecting the first infected individual in "A"
     %%%% First, determining whether the infection "F" occurred during the isolation period
     Lc1=[];
     for ss=1:HB(A(F,3),1)
              cs1=A(F,11)>HB(A(F,3),2*ss) & A(F,11)<HB(A(F,3),(2*ss+1));
              Lc1(ss)=cs1;
     end
     if sum(Lc1)>0           % If the infection source is in isolation, it cannot continue to infect others.
         Exp=exprnd(A(F,9)/A(F,5));
         if A(F,(A(F,12)+12))+Exp<=A(F,10)   % Updating the data when the next infection time of
                                              % the infectious source is earlier than the discovery time
                 A(F,12)=A(F,12)+1;
                 A(F,(A(F,12)+12))=A(F,(A(F,12)+12-1))+Exp;
```

```
            A(F,11)=A(F,(A(F,12)+12));
        else
            A(F,11)=day+1;
    end
else
    xiaoshu=abs(A(F,11)-fix(A(F,11)));          % Determining whether the infection occurred
                                                   at daytime or nighttime.
    binop=binornd(1,p);                          % Whether the next infected person is sick
    wo=[A(F,3),(Home(A(F,3),5)+5)];              % Detecting the specific location of the
                                                     infection source in "Home"
    if xiaoshu<0.5                               % In the daytime
        ra=randperm(nhome);
        raw=ra(1);
        pp=0;
        for hb=1:HB(raw,1)                   % Choosing a family that is not under quarantine
            as=A(F,11)>HB(raw,2*hb) & A(F,11)<HB(raw,(2*hb+1));
            pp=pp+as;
        end
        while pp>0
            ra=randperm(nhome);
            raw=ra(1);
            pp=0;
            for hb=1:HB(raw,1)
                as=A(F,11)>HB(raw,2*hb) & A(F,11)<HB(raw,(2*hb+1));
                pp=pp+as;
            end
        end
        rra=randperm(Home(raw,2));
        col=rra(1)+5;
        xinren=[raw,col];
```

```
        while sum((wo-xinren).^2)==0
                ra=randperm(nhome);
                raw=ra(1);
                pp=0;
                for hb=1:HB(raw,1)
                        as=A(F,11)>HB(raw,2*hb) & A(F,11)<HB(raw,(2*hb+1));
                        pp=pp+as;
                end
                while pp>0
                        ra=randperm(nhome);
                        raw=ra(1);
                        pp=0;
                        for hb=1:HB(raw,1)
                                as=A(F,11)>HB(raw,2*hb) &
                                    A(F,11)<HB(raw,(2*hb+1));
                                pp=pp+as;
                        end
                end
                rra=randperm(Home(raw,2));
                col=rra(1)+5;
                xinren=[raw,col];
        end
    else
        raw=A(F,3);                      % Choosing a susceptible person in "Home"
        rra=randperm(Home(raw,2));
        col=rra(1)+5;
        xinren=[raw,col];
        if Home(A(F,3),2)>1
                while sum((wo-xinren).^2)==0
                        rra=randperm(Home(raw,2));
```

```
                    col=rra(1)+5;
                    xinren=[raw,col];
                end
            end
        end
        xinren=[raw,col];
        Home(A(F,3),5)=xinren(2);
        if sum((wo-xinren).^2)~=0 & Home(xinren(1),xinren(2))~=10 &
           Home(xinren(1),xinren(2))~=20
            Home(xinren(1),3)=Home(xinren(1),3)+binop;
            Home(xinren(1),4)=Home(xinren(1),4)+1-binop;
            Home(xinren(1),xinren(2))=20*binop+10*(1-binop);
            A((lengthA+1),1)=lengthA+1;
            A((lengthA+1),2)=F;
            A((lengthA+1),3)=xinren(1);
            A((lengthA+1),4)=binop;
            A((lengthA+1),5)=R1(unidrnd(nhome))*binop+R0*(1-binop);
            A((lengthA+1),6)=A(F,11);
            A((lengthA+1),7)=incubation(unidrnd(nhome));
            A((lengthA+1),8)=A((lengthA+1),6)+A((lengthA+1),7);
            A((lengthA+1),9)=binop*med(unidrnd(nhome))+(1-binop)*infect0(unidrnd(nhome));
            A((lengthA+1),10)=A((lengthA+1),8)+A((lengthA+1),9);
            binoq=binornd(1,q);                    % Whether the infection source or close
                                                      contacts can be detected
            pai=paicha(unidrnd(nhome));
            if A(F,4)==1                           % If the infected person is sick
                if binoq==1
                    pj=(A(F,10)+pai)<A((lengthA+1),10) &
                       A(F,10)+pai)>A((lengthA+1),8);
                    A((lengthA+1),10)=pj*(A(F,10)+pai)+(1-pj)*A((lengthA+1),10);
```

```
                HA(lengthA+1)=pj;
        end
end
if A((lengthA+1),4)==1 | (A((lengthA+1),4)==0 & HA(lengthA+1)==1)
  % Adjusting the isolation period of the family
        Lc=[];
        for st=1:HB(A((lengthA+1),3),1)
                cs=A((lengthA+1),10)>HB(A((lengthA+1),3),2*st) &
                   A((lengthA+1),10)<HB(A((lengthA+1),3),(2*st+1));
                Lc(st)=cs;
        end
        if sum(Lc)>0                  % "sum(Lc)>0" indicates that the detection
                                        time is during the isolation period.
                fi=find(Lc==1);
                HB(A((lengthA+1),3),(2*max(fi)+1))=A((lengthA+1),10)+14;
           else
                Zc=[];
                for sk=1:(HB(A((lengthA+1),3),1)-1)
                          ct=A((lengthA+1),10)>HB(A((lengthA+1),3),(2*sk+1))
                           & A((lengthA+1),10)<HB(A((lengthA+1),3),(2*sk+2));
                          Zc(sk)=ct;
                end
                if sum(Zc)>0
                          zc=Zc(find(Zc==1));
                          HB(A((lengthA+1),3),(2*zc+2))=A((lengthA+1),10);
                    else if A((lengthA+1),10)<HB(A((lengthA+1),3),2)
                           HB(A((lengthA+1),3),2)=A((lengthA+1),10);
                    end
                    if A((lengthA+1),10)>
                        HB(A((lengthA+1),3),(2*HB(A((lengthA+1),3),1)+1)) &
```

```
                    HB(A((lengthA+1),3),(2*HB(A((lengthA+1),3),1)+1))>0
                        HB(A((lengthA+1),3),1)=HB(A((lengthA+1),3),1)+1;
                        HB(A((lengthA+1),3),2*HB(A((lengthA+1),3),1))=
                        A((l engthA+1),10);
                        HB(A((lengthA+1),3),(2*HB(A((lengthA+1),3),1)+1))=
                        A((lengthA+1),10)+14;
                  end
             end
        end
        if HB(A((lengthA+1),3),2)==0
             HB(A((lengthA+1),3),2)=A((lengthA+1),10);
             HB(A((lengthA+1),3),3)=A((lengthA+1),10)+14;
        end
end
HA(lengthA+1)=0;
Exp=exprnd(A((lengthA+1),9)/A((lengthA+1),5));
if (A((lengthA+1),8)+Exp)<A((lengthA+1),10)
          A((lengthA+1),13)=A((lengthA+1),8)+Exp;
          A((lengthA+1),11)=A((lengthA+1),13);
          A((lengthA+1),12)=1;
     else
          A((lengthA+1),11)=day+1;
end
binoq=binornd(1,q);                  % Whether the infection source can be traced back
pai=paicha(unidrnd(nhome));
if A((lengthA+1),4)==1
        if binoq==1
              pj=(A((lengthA+1),10)+pai)<A(F,10) &
                   (A((lengthA+1),10)+pai)>A(F,8);
              A(F,10)=pj*(A((lengthA+1),10)+pai)+(1-pj)*A(F,10);
```

```
                HA(A(F,3))=2*pj;
        end
end
if HA(A(F,3))==2
        Lc=[];
        for st=1:HB(A(F,3),1)
                cs=A(F,10)>HB(A(F,3),2*st) & A(F,10)<HB(A(F,3),(2*st+1));
                Lc(st)=cs;
        end
        if sum(Lc)>0
                fi=find(Lc==1);
                HB(A(F,3),(2*max(fi)+1))=A(F,10)+14;
           else
                Zc=[];
                for sk=1:(HB(A(F,3),1)-1)
                        ct=A(F,10)>HB(A(F,3),(2*sk+1)) &
                           A(F,10)<HB(A(F,3),(2*sk+2));
                        Zc(sk)=ct;
                end
                if sum(Zc)>0
                        zc=Zc(find(Zc==1));
                        HB(A(F,3),(2*zc+2))=A(F,10);
                    else if A(F,10)<HB(A(F,3),2)
                        HB(A(F,3),2)=A(F,10);
                    end
                    if A(F,10)>HB(A(F,3),2*HB(A(F,3),1)) &
                       HB(A(F,3),2*HB(A(F,3),1))>0
                        HB(A(F,3),1)=HB(A(F,3),1)+1;
                        HB(A(F,3),2*HB(A(F,3),1))=A(F,10);
                        HB(A(F,3),(2*HB(A(F,3),1)+1))=A(F,10)+14;
```

```
                        end
                    end
                end
                if HB(A(F,3),2)==0
                    HB(A(F,3),2)=A(F,10);
                    HB(A(F,3),3)=A(F,10)+14;
                end
            end
            HA(A(F,3))=0;
        end
        Exp=exprnd(A(F,9)/A(F,5));
        if A(F,(A(F,12)+12))+Exp<=A(F,10)
            A(F,12)=A(F,12)+1;
            A(F,(A(F,12)+12))=A(F,(A(F,12)+12-1))+Exp;
            A(F,11)=A(F,(A(F,12)+12));
          else
            A(F,11)=day+1;
        end
      end
suma=sum(A(:,11)<day);
lengthA=size(A,1);
end
%%%% Calculating the AIIs
a08=A(find(A(:,4)==0),8);
a09=A(find(A(:,4)==0),10);
A0=[a08,a09];
%%%% Calculating the patients
a18=A(find(A(:,4)==1),8);
a19=A(find(A(:,4)==1),10);
A1=[a18,a19];
```

```
    Sumw=[];
    for jk=1:day
        sumw=0;
        for ik=1:length(a08)
            pg=jk>A0(ik,1) & jk<A0(ik,2);
            sumw=sumw+pg;
        end
        Sumw(jk)=sumw;
    end
    Sump=[];
    for jk=1:day
        sump=0;
        for ik=1:length(a18)
            pg=jk>A1(ik,1) & jk<A1(ik,2);
            sump=sump+pg;
        end
        Sump(jk)=sump;
    end
    Infections=[Infections;Sumw];
    Patients=[Patients;Sump];
end
Pi75=prctile(Infections,75);
Pi50=prctile(Infections,50);
Pi25=prctile(Infections,25);

Pp75=prctile(Patients,75);
Pp50=prctile(Patients,50);
Pp25=prctile(Patients,25);
```

```
    Storei=[Storei;Pi75;Pi50;Pi25];
    Storep=[Storep;Pp75;Pp50;Pp25];
end
%%%%%%%%%%%%%%%%%%%%%%%%%%%%%%%%%% Drawing the AIIs when R0 has different values
%%R0=0.8
P08=Storei(1:3,:);
hold on
t=1:day;
x=[t,flip(t)];
yp=[L1,flip(L3)];
f1=fill(x, yp, 'b','facealpha',0.4);
p1=plot(t,L2,'-b','LineWidth',2)

%%R0=1.2
P12=Storei(4:6,:);
L1=P12(1,:);L2=P12(2,:);L3=P12(3,:);
x=[t,flip(t)];
yp=[L1,flip(L3)];
f2=fill(x, yp, 'g','facealpha',0.4);
p2=plot(t,L2,'-g','LineWidth',2)

%%R0=1.6
P16=Storei(7:9,:);
L1=P16(1,:);L2=P16(2,:);L3=P16(3,:);
x=[t,flip(t)];
yp=[L1,flip(L3)];
f3=fill(x, yp, 'r','facealpha',0.4);
p3=plot(t,L2,'-r','LineWidth',2)

set(gca,'tickdir','out')
```

```
h=legend([p1,p2,p3],'\fontsize{15}\it R0=0.8','\fontsize{15}\it R0=1.2','\fontsize{15}\it
    R0=1.6','Location','NorthEast')
set(h,'box','off')
axis([0,180,0,1500])
set(gca,'fontsize',16,'fontname','arial');
xticks([0:20:180])
yticks([0:250:1500])
xlabel('days','fontsize',16,'fontname','arial')
ylabel('no. of individuals','fontsize',16,'fontname','arial')

%%%%%%%%%%%%%%%%%%%%%%%%%%%%%%%%%%%%%%%%%%%%%%%%%%%%%%%%%%%%%%%% Drawing the patients when R0 has different values
%%R0=0.8
P08=Storep(1:3,:);
L1=P08(1,:);L2=P08(2,:);L3=P08(3,:);
hold on
t=1:day;
x=[t,flip(t)];
yp=[L1,flip(L3)];
f1=fill(x, yp, 'b','facealpha',0.4);
p1=plot(t,L2,'-b','LineWidth',2)

%%R0=1.2
P12=Storep(4:6,:);
L1=P12(1,:);L2=P12(2,:);L3=P12(3,:);
x=[t,flip(t)];
yp=[L1,flip(L3)];
f2=fill(x, yp, 'g','facealpha',0.4);
p2=plot(t,L2,'-g','LineWidth',2)

%%R0=1.6
```

```
P16=Storep(7:9,:);
L1=P16(1,:);L2=P16(2,:);L3=P16(3,:);
x=[t,flip(t)];
yp=[L1,flip(L3)];
f3=fill(x, yp, 'r','facealpha',0.4);
p3=plot(t,L2,'-r','LineWidth',2)

set(gca,'tickdir','out')
h=legend([p1,p2,p3],'\fontsize{15}\it R0=0.8','\fontsize{15}\it R0=1.2','\fontsize{15}\it
    R0=1.6','Location','NorthEast')
set(h,'box','off')
axis([0,180,0,120])
set(gca,'fontsize',16,'fontname','arial');
xticks([0:20:180])
yticks([0:20:120])
xlabel('days','fontsize',16,'fontname','arial')
ylabel('no. of individuals','fontsize',16,'fontname','arial')
```
